# Chest computed tomography severity score is a reliable predictor of mortality in patients with chronic obstructive pulmonary disease co-infected with COVID-19

**DOI:** 10.1186/s40001-023-01336-8

**Published:** 2023-09-15

**Authors:** Yalda Alipour Khabir, Sevda Alipour Khabir, Hassan Anari, Bahman Mohammadzadeh, Saeed Hoseininia, Mohammad Reza Aslani

**Affiliations:** 1 Students Research Committee, School of Medicine, Ardabil University of Medical Sciences, Ardabil, Iran; 2 Department of Radiology, Faculty of Medicine, Ardabil University of Medical Sciences, Ardabil, Iran; 3 Lung Diseases Research Center, Ardabil University of Medical Sciences, Ardabil, Iran; 4 Department of Physiology, Faculty of Medicine, Ardabil University of Medical Sciences, Ardabil, Iran

**Keywords:** COVID-19, Chronic obstructive pulmonary disease, Computed tomography

## Abstract

**Background:**

Coronavirus disease 2019 (COVID-19) pandemic is considered a global health crisis. The data related to chronic obstructive pulmonary disease (COPD) patients with COVID-19 are incomplete, especially the findings of the chest computed tomography (CT). The aim of the current study was to investigate the severity of the disease of COVID-19 in patients with COPD based on CT severity score and to evaluate its predictive power in the mortality of patients.

**Methods:**

In a retrospective study, demographic, clinical, and CT scan findings of COPD patients with COVID-19 were extracted from March 2020 to February 2022. CT severity score was determined based on the extent and nature of involvement of lungs in CT scan findings. By performing receiver operating characteristics (ROC) and Kaplan–Meier survival analysis were determined the disease severity and survival probability.

**Results:**

The most frequent radiological findings in chest CT scan included ground glass opacities (89.3%), consolidations (51.8%), crazy-paving pattern (46.4%), and septal thickening (35.7%). The mean CT severity score of deceased patients (34.61 ± 18.73) was significantly higher than recovered patients (16.71 ± 14.01, p < 0.001). Based on the ROC and Kaplan–Meier survival curves, it was revealed that CT severity score was a valuable criteria in the diagnosis of mortality in COPD patients with COVID-19.

**Conclusion:**

The findings of this study revealed that the CT severity scoring in COPD patients with COVID-19 was valuable in identifying poor prognosis, although further studies are needed.

## Background

In December 2019, a novel beta-coronavirus, severe acute respiratory syndrome coronavirus 2 (SARS-CoV-2), emerged in Wuhan city, China. The infectious disease caused by this novel coronavirus was named “coronavirus disease 2019 (COVID-19)” by WHO in February 2020. One month later in March 2020, WHO officially declared the disease a globally pandemic and COVID-19 has been a global health crisis for more than two years [1]. The clinical spectrum of SARS-CoV-2 infection ranges from asymptomatic patients to critically ill ones with acute respiratory distress syndrome and multiorgan dysfunction [2]. Male individuals, elderly people, and patients with comorbidities such as diabetes, hypertension, obesity, and cardiovascular disease are at higher risk of poor COVID-19 outcomes and require more attention [3–7].

Early in the SARS-CoV-2 pandemic there was a growing concern about the outcomes in chronic obstructive pulmonary disease (COPD) patients who developed COVID-19. COPD, a common cause of disability and death worldwide, is a persistent dysfunction of the lung characterized by airflow limitation due to inflammation of the airway and/or alveolar abnormalities. COVID-19 patients with preexisting COPD are among high-risk groups due to various complications. These complications include virus-induced exacerbations, impaired lung function, compromised immune responses, and upregulation of angiotensin-converting enzyme 2 (ACE-2) receptor. ACE-2 receptor facilitates SARS-CoV-2 entry into cells putting these patients at higher risk of COVID-19 infection [8–13].

Chest computed tomography (CT) is an effective modality for COVID-19 diagnosis and monitoring the progression of the disease. Studies have discussed that CT imaging has a sensitivity higher than RT-PCR (98% compared to 71%); therefore, chest CT can be used as a screening tool in suspected cases of COVID-19. According to previous studies, ground glass opacities and consolidations with peripheral and subpleural distribution are two main CT findings in COVID-19 [14–19]. The extent and nature of these findings are predictive of prognosis [15]. There are several studies that use a chest CT-based scoring system to determine the risk of disease deterioration and poor outcomes in patients with COVID-19. These studies have demonstrated that higher scores in patients’ CT are associated with higher mortality and severe forms of COVID-19 [18, 20–24]. However, there are limited data on predictive value of a CT severity score in COVID-19 patients who have preexisting COPD. Taking that into consideration, the aim of the current study was to investigate the severity of the disease of COVID-19 in patients with COPD history based on CT severity score and to evaluate its predictive power in the mortality of patients.

## Methods

This was a single-center retrospective study conducted on COPD patients diagnosed with COVID-19 who were hospitalized in Ardabil Imam Khomeini hospital, northwestern Iran from March 2020 to February 2022. All patients who underwent chest CT scan within 24 h of admission in the radiology department of the hospital, included in this study. The diagnosis of COVID-19 was confirmed with a positive RT-PCR for SARS-CoV-2 based on nasopharyngeal and oropharyngeal swabs. Exclusion was determined by certain conditions: (a) patients with a negative RT-PCR, (b) absence of CT findings in mild patients, (c) inadequate quality of chest CT images for analysis, and (d) age below 18 years. This study was approved by the ethics committee (IR.ARUMS.REC.1400.312).

### Data collection

Data including demographic information, duration of hospitalization, inpatient department, comorbidities, and outcome of the disease (recovered or dead) were obtained from each patient’s electronic medical records. Comorbidities included hypertension, chronic kidney disease, chronic liver disease, diabetes mellitus, and coronary artery disease. Based on disease severity patients divided into 3 groups, moderate, severe, and critical cases. Moderate disease included patients with symptoms and signs of pneumonia such as fever, cough, and dyspnea but no signs of severe pneumonia; severe disease additionally met the following criteria––oxygen saturation at rest ⩽ 93% and arterial blood oxygen partial pressure (PaO_2_)/oxygen concentration (FiO_2_) ⩽ 300 mmHg; and the critical disease included patients who were admitted to ICU, were in need of mechanical ventilation, and had signs of multiorgan failure.

### Chest CT interpretation

All patients’ chest CT images were evaluated by two certified radiologists. Both radiologists were blinded to the patients’ data and the final assessments were made by consensus. Definitions of radiological findings were based on the Fleischner Society recommendations, published in 2008 [25]. In this study we used a chest CT severity scoring system first proposed by Ooi et al.[26] in 2004 for SARS. According to this system, each lung was evaluated in 3 levels: upper (above the carina), middle (below the carina up to the upper limit of the pulmonary vein), and lower (below the inferior pulmonary vein). Each level was evaluated separately. Levels were assessed in both nature and extents of the involvement. For evaluating the nature of the involvement grading was as follows: 1 for no pulmonary involvement; 2 for at least 75% GGO/crazy-paving pattern; 3 for a combination of GGO/crazy-paving pattern and consolidation with less than 75% involvement for each; and 4 for at least 75% consolidation.

The score for evaluating the extent of involvement ranged from 0 to 4 as follows: 0 for no involvement, 1 for 1–24%, 2 for 25–49%, 3 for 50–74%, and 4 for more than 75%. The score for each level was calculated by multiplying these two scores, and final score was determined by adding up the scores at these levels in both lungs (ranging from 0 to 96). When present, other lung abnormalities such as septal thickening, reticulation, air bronchogram, pleural thickening, halo sign, lymphadenopathy, and bronchiectasis were also described. Distribution of pulmonary findings were classified as central, peripheral, or diffuse. Findings were also described as unilateral or bilateral.

### Statistical analysis

Data were investigated with SPSS version 21 and MedCalc version 19.4.1 software. Normally distributed variables are expressed by mean ± standard deviation (SD) and categorical variables by percentages. The t-test was used to compare the continuous variables. As for the categorical variables the chi-squared test was used. For estimating the optimal cut-off score, a Receiver Operating Characteristics (ROC) curve analysis was performed (according to Youden’s index for maximizing sensitivity and specificity). Survival probability for CT severity score was estimated using the means of the Kaplan–Meier curves, with the endpoint being death. Cox proportional hazards regression was performed for both univariate and multivariate analyses. The *P*-value was considered significant when less than 0.05 in all analyses.

## Results

### Patient characteristics and outcome

This study involved 56 patients: 26 (46.4%) men and 30 (53.6%) women, the mean age was 68.01 ± 12.08 years. All patients had an underlying COPD and all were diagnosed with COVID-19. The most frequent comorbidities besides COPD were hypertension (39.3%), diabetes mellitus (21%), and cardiovascular disease (14.3%).

Among patients 60.7% were classified as having moderate disease, 7% as severe and 32.1% as critical. 62.5% of patients were admitted in general wards, while 37.5% were ICU patients. For clinical outcomes, 62.5% of patients recovered from the disease and were discharged, while 37.5% expired. Table 1 shows patients’ characteristics in the survival and non-survival groups.

**Table 1:** Patient characteristics in the survival and non-survival patients

Variables	All patients (*n* = 56)	Survival (*n* = 35)	Non-survival (*n* = 21)	*P*-value
Age				
Mean ± SD (year)	68.01 ± 12.08	65.65 ± 11.37	71.95 ± 12.47	0.77
Sex, N (%)				
Male	26 (46.4)	17 (48)	9 (42)	0.67
Female	30 (53.6)	18 (51)	12 (57)	
Comorbidities, N (%)				
Cardiovascular	8 (14)	5 (14)	3 (14)	1
Myocardial infraction	2 (3)	2 (5)	0	0.26
Heart failure	4 (7)	3 (8)	1 (4)	0.59
Type 2 diabetes	12 (21)	5 (8)	7 (33)	0.09
Hypertension	22 (39)	13 (37)	9 (42)	0.67
Cerebrovascular attack	3 (5)	1 (2)	2 (9)	0.28
Kidney diseases	1 (1)	1 (2)	0	0.43
Hospital stay				
Mean ± SD (day)	8 ± 6	6.2 ± 3.8	11.1 ± 7.6	0.000
Diseases severity, N (%)				0.000
Moderate	34 (60)	32 (91)	2 (9)	
Severe	4 (7)	2 (5)	2 (9)	
Critical	18 (32)	1 (2)	17 (80)	

An independent t-test was used to contrast the statistical differences between non-survival and survival COPD patients with COVID-19

### Chest CT findings

The most frequent radiological findings in chest CT scan included ground glass opacities (89.3%), consolidations (51.8%), crazy-paving pattern (46.4%), and septal thickening (35.7%). CT findings were mostly bilateral (78.6%) and multi-focal (75%) and were distributed peripherally (76.8%). Table 2 summarizes chest CT findings and their distribution in the survival and death groups.

**Table 2:** Chest CT findings in survival and non-survival COVID-19 COPD patients

Nature of the findings	Total patients (*n* = 56) *N* (%)	Survival (*n* = 35) *N* (%)	Non-survival (*n* = 21) *N* (%)	P-value
Ground glass opacities	50 (89)	31 (88)	19 (90)	0.82
Vascular enlargement	5 (8)	1 (2)	4 (19)	0.04
Posterior predilection	21 (37)	15 (42)	6 (28)	0.28
Consolidation	29 (51)	16 (45)	13 (61)	0.24
Linear opacities	9 (16)	5(14)	4 (19)	0.63
Septal thickening	20 (35)	10 (28)	10 (47)	0.15
Reticulation	11 (19)	5 (14)	6 (28)	0.19
Crazy-paving pattern	26 (46)	13 (37)	13 (61)	0.07
Air bronchogram	6 (10)	2 (5)	4 (19)	0.11
Pleural thickening	3 (5)	2 (5)	1 (4)	0.87
Halo sign	3 (5)	3 (8)	0	0.16
Bronchiectasis	6 (10)	2 (5)	4 (19)	0.11
Nodules	5 (8)	4 (11)	1 (4)	0.39
Bronchial wall thickening	5 (8)	1 (2)	4 (19)	0.04
Pleural effusion	8 (14)	4 (11)	4 (19)	0.43
Pericardial effusion	1 (1)	1 (2)	0	0.43
Bilateral findings	44 (78)	25 (71)	19 (90)	0.09
Unilateral findings	9 (16)	8 (22)	1 (4)	0.07
Upper or middle lobe involvement	49 (87.5)	29 (82)	20 (95)	0.17
Lower lobe involvement	45 (80)	28 (80)	17 (80)	0.93
Central location	3 (5)	2 (5)	1 (4)	0.87
Peripheral location	43 (76)	27 (77)	16 (76)	0.93
Central and peripheral location	17 (30)	6 (17)	11 (52)	0.005
Single-focal lesion	6 (10)	5 (14)	1 (4)	0.26
Multi-focal lesion	42 (75)	26 (74)	16 (76)	0.87
Diffuse lesion	25 (44)	13 (37)	12 (57)	0.14

An independent t-test was used to contrast the statistical differences between non-survival and survival patients

### Chest CT severity score

Table 3 shows the CT severity score based on nature of the involvement and extent of the involvement at different levels (upper, middle, and lower levels) of both right and left lungs as well as the whole lung. The mean CT severity score in non-surviving patients (34.61 ± 18.73) was significantly higher than surviving patients (16.71 ± 14.01, *p* < 0.001) (Table 4). In addition, the mean score of patients hospitalized in ICU (36.66 ± 18.33) was significantly higher than that of patients hospitalized in the general department (15.48 ± 12.47, *p* < 0.001).

**Table 3:** Mean ± SD of CT severity scores in each level of the lung

Lung levels	Nature of the involvement	Extent of the involvement	Total score
Right upper level	1.64 ± 1.25	2.35 ± 0.99	4.30 ± 3.48
Right middle level	1.53 ± 1.12	2.35 ± 1.01	4.12 ± 3.25
Right lower level	1.55 ± 1.21	2.32 ± 1.01	4.25 ± 3.70
Total right lung	–	–	12.67 ± 9.44
Left upper level	1.23 ± 1.15	2.12 ± 1.01	3.23 ± 3.23
Left middle level	2.14 ± 0.99	1.32 ± 1.17	3.44 ± 3.17
Left lower level	2.17 ± 1.09	2.17 ± 1.09	4.12 ± 3.97
Total left lung	–	–	10.75 ± 9.29
Total lung	–	–	23.42 ± 18.04

Upper lobe: above the carina, middle lobe: below the carina up to the upper limit of the pulmonary vein, and lower lobe: below the inferior pulmonary vein

**Table 4:** Mean ± SD of CT severity scores in survival and non-survival COVID-19 COPD patients

Lung levels	Survival (*n* = 35)	Non-survival (*n* = 21)	*P*-value
Right upper level	3.17 ± 3.11	6.19 ± 3.31	0.001
Right middle level	2.88 ± 2.59	6.19 ± 3.28	0.0001
Right lower level	3.14 ± 2.73	6.09 ± 4.39	0.003
Total right lung	9.20 ± 7.54	18.47 ± 9.60	0.0001
Left upper level	2.05 ± 2.43	5.19 ± 3.50	0.0001
Left middle level	2.54 ± 2.52	4.95 ± 3.61	0.005
Left lower level	3 ± 3.17	6 ± 4.51	0.005
Total left lung	7.51 ± 7.37	16.14 ± 9.80	0.0001
Total lung	16.71 ± 14.01	34.61 ± 18.73	0.0001

An independent t-test was used to contrast the statistical differences between non-survival and survival COPD patients with COVID-19. Upper lobe: above the carina, middle lobe: below the carina up to the upper limit of the pulmonary vein, and lower lobe: below the inferior pulmonary vein

### Receiver operating characteristics (ROC)

**Fig. 1:**
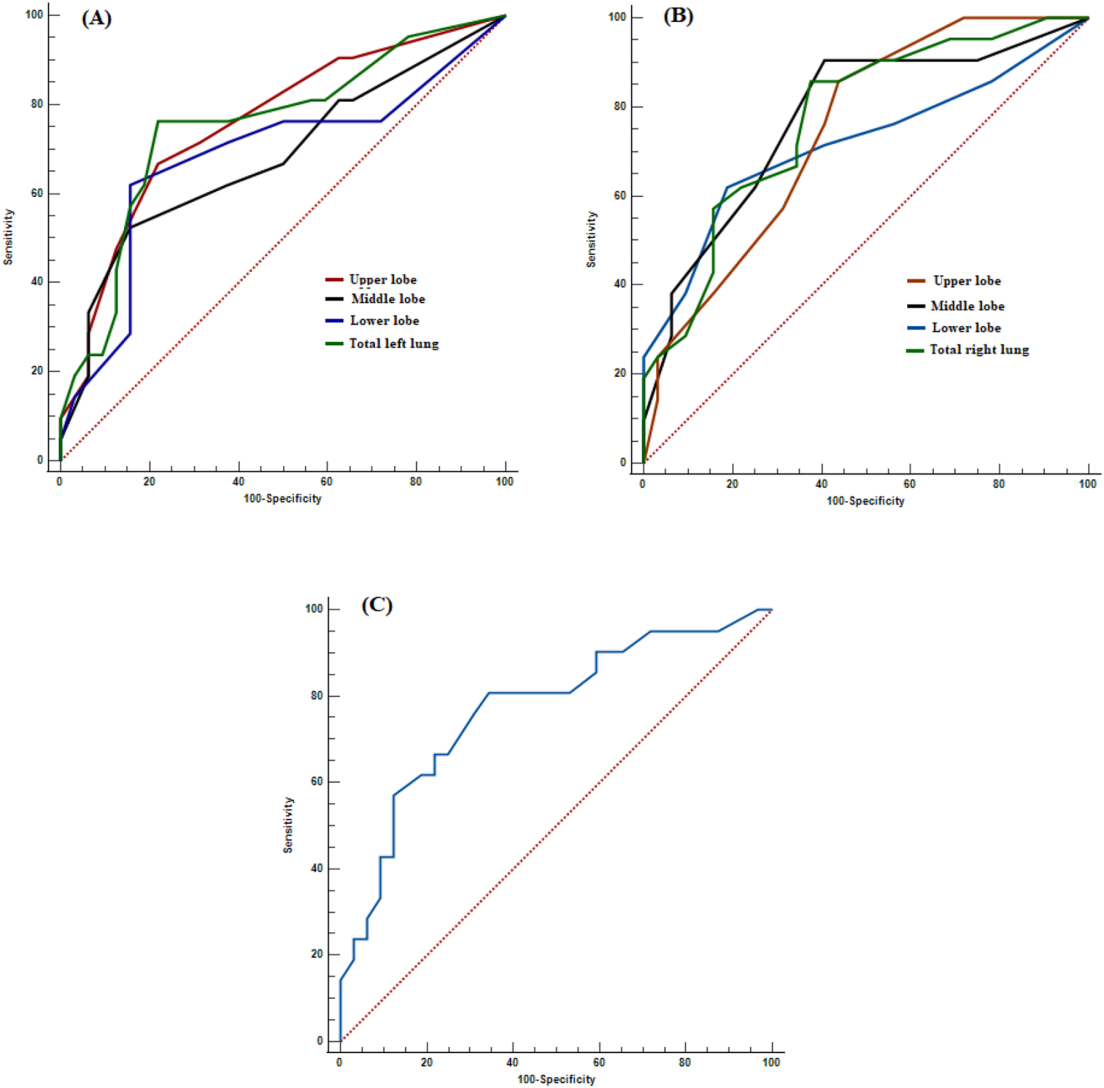
Receiver operating characteristics curve of COPD patients with COVID-19 for CT severity score of **A**: left lung, **B**: right lung, and **C**: total lung. Upper lobe: above the carina, middle lobe: below the carina up to the upper limit of the pulmonary vein, and lower lobe: below the inferior pulmonary vein

Optimal cut-off points observed for CT severity score predicated on the ROC curve for surviving assessment were upper level of left lung (> 3), middle level of left lung (> 4), lower level of left lung (> 4), total level of left lung (> 11), upper level of right lung (> 2), middle level of right lung (> 3), lower level of right lung (> 4), total level of right lung (> 10), and total level of lung (> 18) (Fig. 1A, B, C and Table 5).

**Table 5:** Receiver operating characteristics (ROC) curves and prognostic accuracy of CT severity score in COVID-19 COPD patients

Variables	AUC	95% CI	*p*-Value	Cut-off	Sensitivity	Specificity (%)
Upper level of left lung	0.760	0.623 to 0.866	0.000	> 3	66.6	78.1
Middle level of left lung	0.682	0.539 to 0.803	0.020	> 4	52.3	84.3
Lower level of left lung	0.682	0.540 to 0.803	0.025	> 4	61.9	84.3
Total level of left lung	0.754	0.616 to 0.862	0.000	> 11	76.1	78.1
Upper level of right lung	0.746	0.607 to 0.885	0.000	> 2	85.7	56.2
Middle level of right lung	0.774	0.638 to 0.877	0.000	> 3	90.4	59.3
Lower level of right lung	0.717	0.577 to 0.832	0.005	> 4	61.9	81.2
Total level of right lung	0.773	0.637 to 0.877	0.000	> 10	85.7	62.5
Total level of lung	0.772	0.637 to 0.876	0.000	> 18	80.9	65.6

Upper lobe: above the carina, middle lobe: below the carina up to the upper limit of the pulmonary vein, and lower lobe: below the inferior pulmonary vein

Moreover, significant AUC levels demonstrated in regard to CT severity score were upper level of left lung (0.760), middle level of left lung (0.682), lower level of left lung (0.682), total level of left lung (0.754), upper level of right lung (0.746), middle level of right lung (0.774), lower level of right lung (0.717), total level of right lung (0.773), and total level of lung (0.772) (Fig. 1A, B, C and Table 5).

**Fig. 2:**
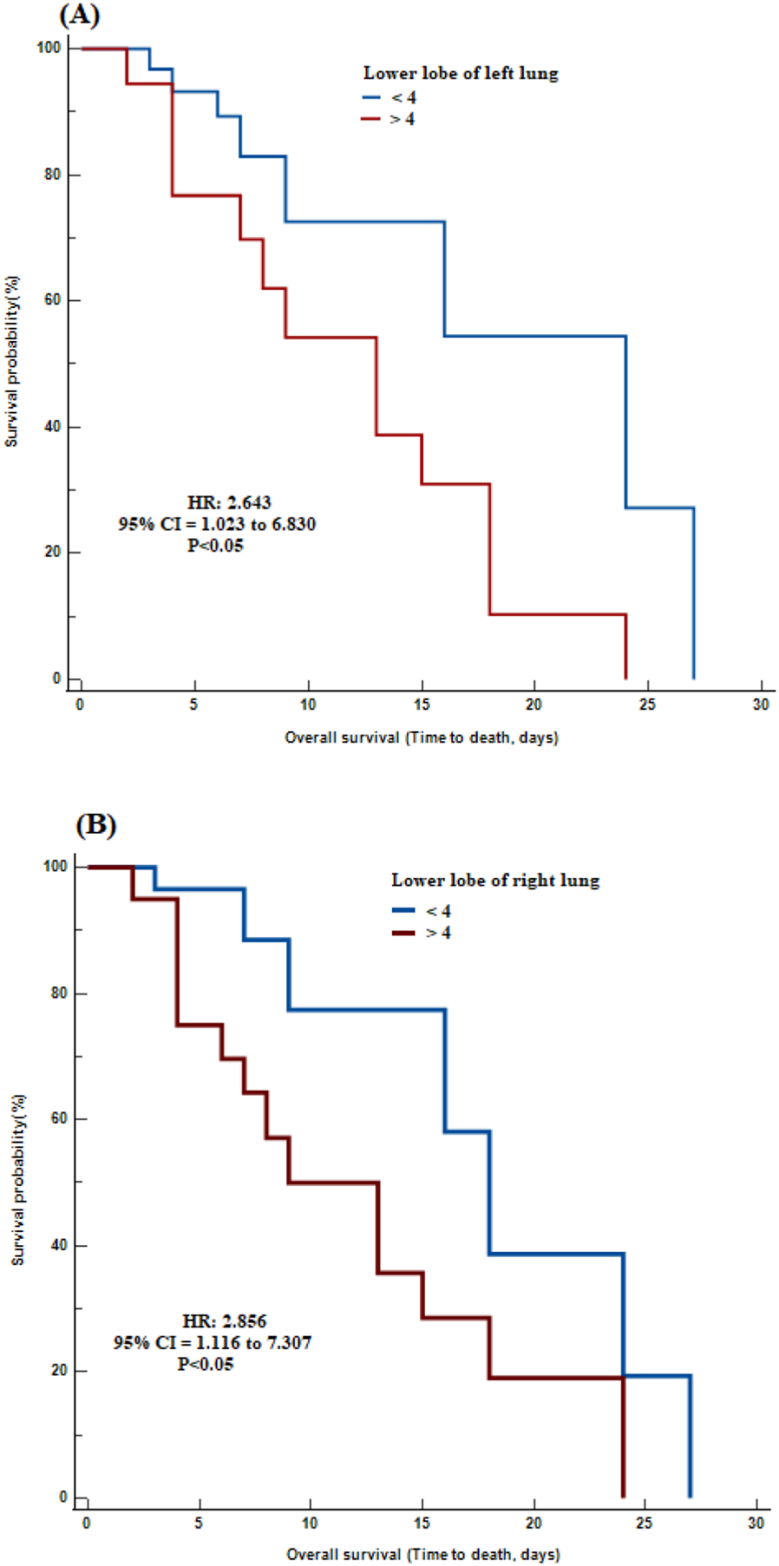
Kaplan–Meier survival curves during hospitalization of COVID-19 COPD patients with different cut-off values of **A**: lower level of left lung and **B**: lower level of right lung. Lower lobe: below the inferior pulmonary vein

The Kaplan–Meier survival curve showed that higher levels of lower level of left lung (HR = 2.643, 95% CI 1.023 to 6.830, *P* < 0.05) (Fig. 2A) and lower level of right lung (HR = 2.856, 95% CI 1.116 to 7.307, *P* < 0.05) (Fig. 2B) were significantly associated with shorter survival period. Multivariate Cox regression models identified that only lower level of right lung (HR = 2.682, 95% CI  1.019 to 7.058, *P* < 0.05) was significantly associated with survival.

## Discussion

The most important findings of the current study were as follows: 1- The mean CT severity score in patients who died was detectably higher than those who recovered. 2- Based on the ROC and Kaplan–Meier survival curves, it was revealed that CT severity score was a valuable criteria in the diagnosis of mortality in COPD patients with COVID-19. 3- Results from multivariate Cox regression model indicated that lower lung lobes severity score were significantly associated with survival.

Many studies have been conducted to identify the role of a quantitative CT scoring system in predicting disease severity in COVID-19 patients. To the best of our knowledge this is the first study that focuses on COPD patients infected by SARS- CoV-2 to evaluate CT findings based on a quantitative scoring system. A previous study used a quantitative CT emphysema score to examine its association with clinical outcome in COPD and COVID-19 patients, which demonstrated that scores higher than 5% are associated with disease severity. However, this study did not include COVID-19 specific radiologic findings in the scoring system [27]. According to current data COPD is not a frequent comorbidity in COVID-19 patients [22, 28]. In contrast, some studies have demonstrated that COPD patients were more susceptible to the critical form of COVID-19 [29, 30]. This can be due to impaired lung function in COPD patients and higher ACE-2 expression in these patients which may facilitate viral entry.

The results of the current study revealed that the mean CT score was higher in the non-survival patients in comparison to the survival group. Patients with severe and critical disease also had higher CT scores than moderate cases. This was in agreement with previous studies that suggested higher CT scores in COVID-19 patients are associated with higher mortality and increase the risk of developing severe and critical types of the disease [21, 31]. In addition, it was found that GGO, a crazy-paving pattern, and consolidations are the most frequent findings in COPD patients with COVID-19 and the findings are more predominant in peripheral and they are mostly bilateral, which was consistent with recent studies [14, 16, 17, 19].

During the coronavirus pandemic, the use of different indicators to diagnose the disease prognosis in patients with COVID-19 has been of interest. In our previous study, systemic inflammation indices were used to diagnose the severity of the disease in COVID-19 patients [32–35]. On the other hand, the effectiveness of severity score in patients with COVID-19 has been reported in some studies [18, 36–39]. The results of the current study showed that CT severity score was a valuable measure in diagnosing the severity of the disease in COPD patients infected with SARS-CoV-2. Although all the levels of the right and left lungs (upper, middle, and lower) based on ROC and Kaplan–Meier curves were useful in diagnosing the mortality of patients, the lower levels of the lungs were very efficient.

To our best knowledge, multivariate Cox regression analysis showed that among the different levels of the right and left lung (upper, middle, and lower), the lower level of the right lung remained with survival. It seems that in COPD patients with COVID-19, the involvement of the lower parts of the lungs is associated with a poor prognosis of the disease, which requires further studies.

The limitations of the study were as follows: 1- This study was conducted retrospectively and in a single center. 2- The sample size for the evaluation of COPD patients with COVID-19 was moderate, and a large number of patients is required for more detailed investigations. 3- Although the CT severity score of patients at admission is used to determine the prognosis of the disease, but each patient may be hospitalized with a different severity of the disease. 4- Different stages of COPD may have influenced the results of the study, which could not be evaluated due to the lack of spirometry findings. 5- Medicines used by patients to treat COPD before hospitalization could not be reported due to lack of registration.

## Conclusion

COPD patients are particularly vulnerable to SARS-CoV-2 infection as a result of their specific treatments and accompanying illnesses. Establishing the intensity of the disease at the start of hospitalization in COPD patients can manage their treatment effectively. The current study demonstrated that the CT severity scoring system can be used as a beneficial tool for estimating disease severity and predict prognosis in COVID-19 COPD patients. Interestingly, the lower lobes of lung involvement showed an excellent predictive power for mortality rates in the case of COPD patients infected with COVID-19.

## Data Availability

The data sets used and/or analyzed during the current study are available from the corresponding author on reasonable request.

## Abbreviations

ACE-2: Angiotensin-converting enzyme 2
COPD: Chronic obstructive pulmonary disease
CT: Computed tomography
COVID-19: Coronavirus disease 2019
ROC: Receiver operating characteristics
SARS-CoV-2: Severe acute respiratory syndrome coronavirus 2
